# Largely different carotenogenesis in two pummelo fruits with different flesh colors

**DOI:** 10.1371/journal.pone.0200320

**Published:** 2018-07-09

**Authors:** Fuhua Yan, Meiyan Shi, Zhenyu He, Lianhai Wu, Xianghua Xu, Min He, Jiajing Chen, Xiuxin Deng, Yunjiang Cheng, Juan Xu

**Affiliations:** 1 Key Laboratory of Horticultural Plant Biology, Ministry of Education, Wuhan, Hubei, P.R. China; 2 Forestry Science Academy of Lishui, Lishui, Zhejiang, P.R. China; Beijing Forestry University, CHINA

## Abstract

Carotenoids in citrus fruits have health benefits and make the fruits visually attractive. Red-fleshed ‘Chuhong’ (‘CH’) and pale green-fleshed ‘Feicui’ (‘FC’) pummelo (*Citrus maxima* (Burm) Merr.) fruits are interesting materials for studying the mechanisms of carotenoid accumulation. In this study, particularly high contents of linear carotenes were observed in the albedo tissue, segment membranes and juice sacs of ‘CH’. However, carotenoids, especially β-carotene and xanthophylls, accumulated more in the flavedo tissue of ‘FC’ than in that of ‘CH’. Additionally, the contents of other terpenoids such as limonin, nomilin and abscisic acid significantly differed in the juice sacs at 150 days postanthesis. A dramatic increase in carotenoid production was observed at 45 to 75 days postanthesis in the segment membranes and juice sacs of ‘CH’. Different expression levels of carotenogenesis genes, especially the ζ-carotene desaturase (*CmZDS*), β-carotenoid hydroxylase (*CmBCH*) and zeaxanthin epoxidase (*CmZEP*) genes, in combination are directly responsible for the largely different carotenoid profiles between these two pummelo fruits. The sequences of eleven genes involved in carotenoid synthesis were investigated; different alleles of seven of eleven genes might also explain the largely different carotenogenesis observed between ‘CH’ and ‘FC’. These results enhance our understanding of carotenogenesis in pummelo fruits.

## Introduction

**Fig 1:**
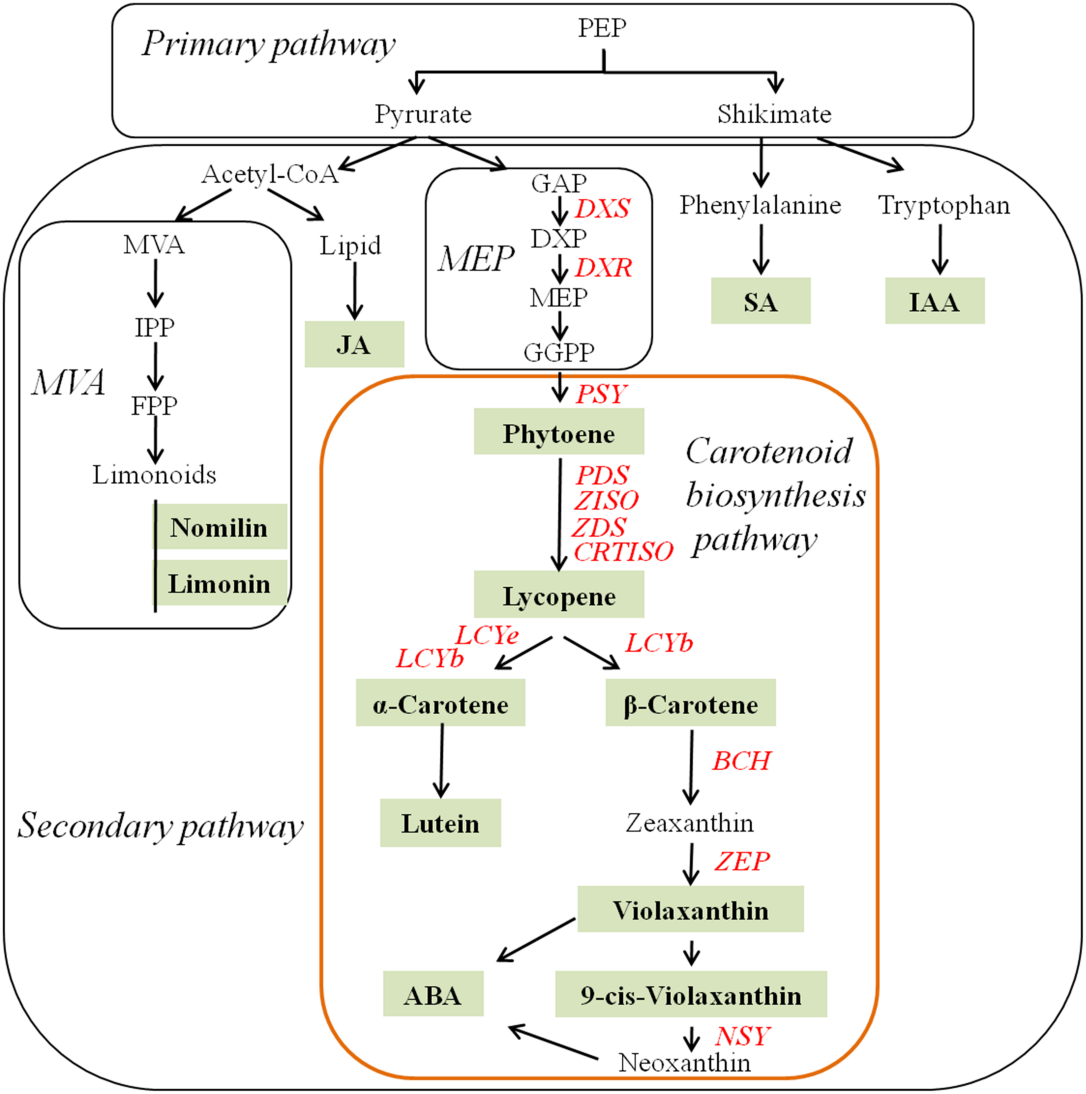
Diagram of metabolic pathways involved in this study. Note: Acetyl-CoA: acetoacetyl-coenzyme A. *BCH*: β-carotene hydroxylase gene. *CCS*: chromoplast-specific lycopene β-cyclase gene. *CRTISO*: carotenoid isomerase gene. DXP: 1-deoxy-D-xylulose-5-phosphate. *CmDXR*: 1-deoxy-D-xylulose-5-phosphate reductoisomerase gene. *DXS*: 1-deoxy-D-xylulose-5-phosphate synthase gene. FPP: farnesyl pyrophosphate. GAP: D-glyceraldehyde-3-phosphate. GGPP: geranylgeranyl diphosphate. IAA: indole-3-acetic acid. IPP: isopentenyl pyrophosphate. JA: jasmonic acid. *LCYb*: lycopene β-cyclase gene. *LCYe*: lycopene ε-cyclase gene. Lipid: lipid (pathway). MVA: mevalonic acid (pathway). MEP: methylerythritol 4-phosphate (pathway). *NSY*: neoxanthin synthase gene. *PDS*: phytoene desaturase gene. PEP: phosphoenolpyruvate. *PSY*: phytoene synthase gene. SA: salicylic acid. *ZDS*: ζ-carotene desaturase gene. *ZEP*: zeaxanthin epoxidase gene. *ZISO*: 15-*cis*-ζ-carotene isomerase gene. The red and italic abbreviations denote genes investigated in this study. The green in the filled frames denote compounds detected in this study.

Carotenoids, especially lycopene, contribute to the red-fleshed color of many plants, such as tomato (*Solanum lycopersicum*) [1], papaya (*Carica papaya*) [2, 3], red carrot (*Daucus carota*) [4], watermelon (*Citrullus lanatus*) [5], pink guava (*Psidium guajava*) [6], gac (*Momordica cochinchinensis*) [7], and red-fleshed citrus (*Citrus*) [8]. These red-colored fruit fleshes have health benefits and are visually attractive. Recently, the mechanism of red-fleshed pigmentation due to carotenoids has attracted increasing amounts of research attention, and relevant candidate genes have been verified in papaya [2], carrot [9] and tomato [10]. Recently, the composition and content of carotenoids in red-fleshed citrus were reported to largely differ from those in pale-fleshed/orange-fleshed citrus [11, 12, 13]. However, owing to the lack of records concerning the breeding history of some red-fleshed citrus cultivars, the naturally occurring highly hybridized genetic background and the long juvenile phase of citrus seedlings [14], the mechanism of carotenogenesis in red-fleshed citrus remains largely unknown. The cyclization of lycopene is a central branch point in the carotenoid biosynthesis pathway (Fig 1). Carotenoids have two main branches: α- and β-branches. β-Branches are common, while α-branches rarely occur in citrus fruit. Compared with β-branches, α-branches are more dominant in the leaves of almost every plant species, producing α-carotene and lutein [15]. The alternative β-branches produce β-carotene, zeaxanthin, violaxanthin, and neoxanthin [16]. Abscisic acid (ABA) is a product of the latter branch (Fig 1) and influences upstream carotenoids as a feedback regulator [17]. In addition, indole-3-acetic acid (IAA), jasmonic acid (JA), and salicylic acid (SA) have been reported to influence carotenoid levels to some extent [18].

Carotenoids accumulate in almost all the tissues of citrus fruits. Specifically, carotenoid accumulation in the flavedo and juice sacs (JS) of fruits has been studied extensively [19, 20, 21, 22]. To our knowledge, 9-*cis*-violaxanthin, violaxanthin, lutein, β-cryptoxanthin, α-carotene, β-carotene, phytofluene and phytoene have been detected in the flavedo of citrus fruits [20, 21, 22]. The total carotenoid contents in the flavedo decrease during fruit development, as lutein, α-carotene and β-carotene all decrease [21, 22]. However, in the JS of citrus fruits, the carotenoid profiles largely vary among different varieties. Orange-fleshed mandarins (*Citrus reticulata*) mainly accumulate 9-*cis*-violaxanthin, β-cryptoxanthin and β-carotene [20, 23]. Interestingly, red-fleshed traits have been observed in some pummelos (*Citrus maxima*) [11, 20, 24], grapefruits (*Citrus paradisi*) [21] and sweet oranges (*Citrus sinensis*) [13, 20, 22, 25]; colorless phytoene, an abundance of red-colored lycopene and orange-colored β-carotene have been reported [22]. Moreover, the total carotenoids in the flesh of citrus fruits generally increase during fruit development [21, 22, 24]. Limonin and nomilin are other terpenoids in citrus and are produced from the mevalonic acid (MVA) biosynthesis pathway (Fig 1). At the mature stage, the high-carotenoid juice sacs of red-fleshed ‘Chuhong’ (‘CH’; *Citrus maxima*) pummelo fruits accumulate an abundance of limonin and nomilin, while the low-carotenoid JS of pale green-fleshed ‘Feicui’ (‘FC’) pummelo fruits are associated with low limonin and nomilin accumulations [11].

The 1-deoxy-D-xylulose-5-phosphate synthase (*DXS*) gene and 1-deoxy-D-xylulose-5-phosphate reductoisomerase (*DXR*) gene are critical in the methylerythritol 4-phosphate (MEP) biosynthesis pathway, which is a well-known pathway that is upstream of the carotenoid biosynthesis pathway [18]. The phytoene synthase (*PSY*) gene has been demonstrated to be a rate-limiting gene involved in carotenoid accumulation [26, 27, 28]. All-*trans*-lycopene is synthesized from phytoene via four steps, and the enzymes coded by the phytoene desaturase (*PDS*) gene, ζ-carotene isomerase (*ZISO*) gene, ζ-carotene desaturase (*ZDS*) gene and carotenoid isomerase (*CRTISO*) gene together with light-mediated isomerization are involved in the process [29, 30]. In addition, the lycopene β-cyclase (*LCYb*) gene was thought to be an important enzyme affecting the accumulation of lycopene [3, 21, 31, 32] (Fig 1). The enzyme coded by the lycopene ε-cyclase (*LCYe*) gene cyclizes lycopene twice to form α-carotene and is the first enzyme of the α-branch. The β-carotenoid hydroxylase (*BCH*), zeaxanthin epoxidase (*ZEP*) and neoxanthin synthase (*NSY*) genes are located downstream of the carotenoid pathway (Fig 1). Compared with that in pale yellow-fleshed ‘Anliu’ (*Citrus sinensis* cv. Anliu) sweet orange, the accumulation of lycopene in its red-fleshed mutant ‘Hong Anliu’ has been attributed to the upregulation of upstream genes and the downregulation of downstream genes [22]. Expression levels of genes involved in the carotenoid pathway have been widely studied [13, 21, 22], although little sequence information has been reported for citrus, especially with respect to the genes involved in the whole carotenoid pathway [13, 33].

**Fig 2:**
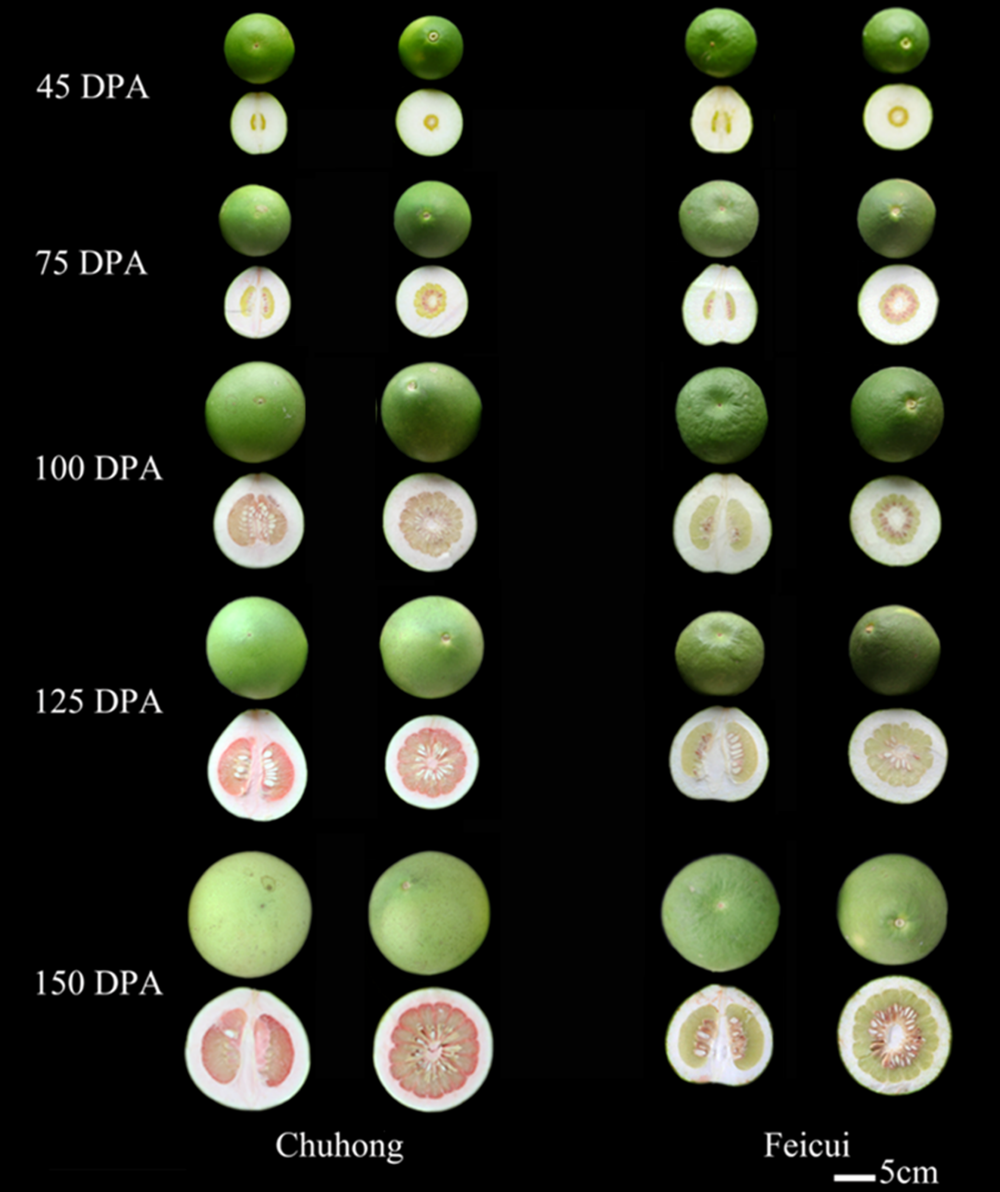
Developmental stages of ‘Chuhong’ and ‘Feicui’ pummelo (*Citrus maxima*) fruits. Note: DPA: days postanthesis. The white ruler shown here is 5 cm in total length.

‘CH’ and ‘FC’ are excellent pummelo cultivars selected from local sources in Lishui, Zhejiang Province, but their origins are unknown. Their fruits are similar in size and shape but present different flesh colors (Fig 2). We previously reported the tissue-specific pattern of carotenoids, the synthesis of limonoid aglycones, and the largely different volatile terpenoid profiles in the mature pummelo fruits of both cultivars [11]. However, the metabolites of whole developmental stages and carotenogenesis based on the expression levels of carotenogenesis genes have not been elucidated. In this study, the carotenoid contents in the fruit tissues of ‘CH’ and ‘FC’ at five developmental stages were investigated. Furthermore, the expression levels of 13 carotenogenesis genes were analyzed, and 11 of those genes were cloned and sequenced. High expression levels of *CmZDS*, *CmBCH* and *CmZEP* in the segment membranes (SMs) and JS of ‘CH’ and different alleles of 7 genes in the JS might be directly responsible for the largely different carotenogenesis between the ‘CH’ and ‘FC’ pummelo fruits.

## Materials and methods

All protocols in this manuscript share the same protocols, 10.17504/protocols.io.qsjdwcn.

### Materials and reagents

‘CH’ and ‘FC’ pummelo (*Citrus maxima*) fruits exhibiting typical characters at each corresponding fruit development stage were collected from the scientific research orchard of the Forestry Science Academy of Lishui, Zhejiang Province, in 2011. The fruits were collected at 45, 75, 100, 125 and 150 days postanthesis (DPA) (Fig 2). The fruits of ‘CH’ and ‘FC’ were commercially mature between 125 DPA and 150 DPA. The flavedo, albedo, SMs and JS of the fruits were carefully dissected [22]. The JS of Huanong Red (HR, red-fleshed), Chandler (QDC, red-fleshed), Hirado Buntan (HB, pink-fleshed), Wubu Red-fleshed (WBH, red-fleshed), Thai (T, pale yellow-fleshed), Kao Pan (KP, pale green-fleshed), Fenghuang (FH, pale green-fleshed) and Acidless (WS, pale green-fleshed) pummelo fruits at full maturity were collected from the National Citrus Breeding Center at Huazhong Agricultural University. Three biological replicates for each sample were prepared, with each biological replicate consisting of 6–8 fruits.

All the samples were treated with liquid nitrogen and then stored at –80°C. Some of them were lyophilized in a Heto Lyolab 3000 (Heto-Holten A/S, Allerød, Denmark) and homogenized to a powder in liquid nitrogen for the extraction of metabolites.

### Extraction and determination of carotenoids

Carotenoids were extracted from 1.0 g of lyophilized samples and then measured in accordance with the methods of Liu *et al*. [22] and Lee [34]. The carotenoids were identified by comparing the retention times and the absorption spectra with those of authentic standards and were quantified by peak areas. The peak areas were converted to concentrations by comparisons with the authentic standards of known concentrations measured by high-performance liquid chromatography (HPLC). Authentic carotenoid standards of antheraxanthin, α-carotene, β-cryptoxanthin, all-*trans*-lutein, all-*trans*-violaxanthin and phytoene were purchased from CaroteNature (Lupsingen, Switzerland), while those of β-carotene and all-*trans*-lycopene together with standards of limonin and nomilin were purchased from Sigma Co. Ltd. (St. Louis, MO, USA).

### Extraction and determination of limonin and nomilin in limonoid aglycones

In accordance with the methods of Li *et al*. [35], 3.0 g of lyophilized powder was extracted with 50 mL of dichloromethane via a FexIKAvarioControl system (IKA-Werke GmbH & Co. KG, Staufen, Germany). The extraction solution was collected and then dried under vacuum in a 5301 concentrator (Eppendorf, Hamburg, Germany) after 15 cycles of Soxhlet extraction (approximately 3 h), after which 1 mL of acetonitrile was ultimately added. Prior to the HPLC analysis, 1 mL of sample tissue was filtered through a micropore film filter (0.22 μm).

With respect to HPLC analyses, the same instrument was used to analyze the carotenoids, while a C_18_ HPLC column (4.6×150 mm, 5 μm, Agilent, USA) was used for limonin and nomilin separation. For quantification, an isocratic elution model was applied with acetonitrile:10% methanol (40:60, v/v) at an elution speed of 1 mL/min and an injection volume of 20 μL (Manners, 2007). Both limonoids were detected at a wavelength of 210 nm under ambient temperature.

### Extraction and determination of phytohormones

In accordance with the methods of Pan *et al*. [36] and Ding *et al*. [37], phytohormone extractions were carried out with 50 mg of lyophilized powder in 500 μL of chilled buffer (isopropanol:water:concentrated hydrochloric acid = 100:50:0.1 v/v/v). After the mixture was incubated at 0°C for more than 12 h and after another 500 μL of chilled buffer was added, the mixture was shaken at a speed of 100 g for 1 h at 4°C. Afterward, 2 mL of chilled dichloromethane was added to the sample, after which the sample was shaken for 1 h at 4°C. The samples were subsequently centrifuged at 12,000 g for 10 min at 4°C to form two phases. The lower phase was collected and concentrated using a nitrogen evaporator. The samples were redissolved in 0.15 mL of methanol and then centrifuged at 12,000 g for 15 min at 4°C, after which 20 μL of the supernatant of each sample was filtered through 0.22-μm micropore filters for injection into a reverse-phase C18 Gemini HPLC column for HPLC-electrospray ionization-tandem mass spectrometry (HPLC-ESI-MS/MS) analysis.

In accordance with the methods of Ma *et al*. [38], the phytohormone extracts were separated with HPLC (Agilent 1100, Agilent Technologies, Palo Alto, CA, USA) and measured via HPLC-ESI-MS/MS (API 3000 mass spectrometer, Applied Biosystems, Foster City, CA, USA), and the MS/MS conditions of each analysis were set in accordance with the methods of Pan *et al*. [36].

Authentic standards of IAA, ABA, SA and JA were purchased from OlChemIm (OlChemIm, Olomouc, Czech Republic).

### RNA extractions and qRT-PCR analysis

In accordance with the methods of Gao *et al*. [39], total RNA was extracted and purified from the tissues of pummelo fruits. To avoid genomic DNA contamination, the total RNA was then treated with DNase I at 37°C for 1 h. The concentration and purity of the total RNA were then determined via a spectrophotometer (Thermo-Fisher Scientific, Wilmington, DE, USA). The transcription levels of *CmDXS* (encoding 1-deoxy-D-xylulose-5-phosphate synthase), *CmDXR* (encoding 1-deoxy-D-xylulose-5-phosphate reductoisomerase), *CmPSY* (encoding phytoene synthase), *CmPDS* (encoding phytoene desaturase), *CmZISO* (encoding ζ-carotene isomerase), *CmZDS* (encoding ζ-carotene desaturase), *CmCRTISO* (encoding carotenoids isomerase), *CmLCYb1* (encoding lycopene β-cyclase gene1), *CmLCYB2* (encoding lycopene β-cyclase gene2), *CmLCYe* (encoding lycopene ε-cyclase gene), *CmBCH* (encoding β-carotene hydroxylase), *CmZEP* (encoding zeaxanthin epoxidase) and *CmNSY* (encoding neoxanthin synthase) were examined via quantitative real-time PCR (qRT-PCR) analysis. The primer sequences of the 8 genes above and of an endogenous control gene (*Actin*) were designed in accordance with the methods of Liu *et al*. [22], while those of *CmDXS*, *CmDXR*, *CmPSY* and *CmZISO*, which are listed in S1 Table, were designed with Primer Premier 5 (Premier, Canada).

Reverse transcription was performed as described by Liu *et al*. [22], while qRT-PCR was performed using an ABI 7500 Real Time System (PE Applied Biosystems, Foster City, CA, USA) in accordance with the methods of Liu *et al*. [22]. For each sample, the expression level of each biological replicate was determined by the mean value of at least three technical replicates. The mean threshold cycle (*Ct*) and the standard deviation (SD) for each sample were obtained in accordance with the methods of Gao *et al*. [39].

### Amplification and sequencing of gene alleles

To amplify the whole coding sequences of the eleven genes identified mainly from the JS of both pummelo fruits, primers were designed in accordance with the methods of Liu *et al*. [22] and Gao *et al*. [39] or via Primer Premier 5; the primers are listed in S2 Table. The whole coding sequences of the *CmDXS* alleles could not be amplified, possibly due to their low expression levels in all fruit tissues. The whole coding sequences of at least 10 clones for each carotenogenesis gene were sequenced from the cDNA mainly from the JS in each pummelo fruit. In particular, with respect to *CmPSY*, *CmLCYb1*, *CmLCYb2*, *CmLYCe* and *CmNSY*, nearly 50 clones of each gene in each pummelo fruit were obtained. However, due to the difficulties in obtaining new clones, only 15 clones of *CmZEP* were obtained for ‘FC’.

A Phanta^®^ Max Super-Fidelity DNA Polymerase Kit (Vazyme Biotech Co., Ltd) was used for allele amplification. The PCR products were electrophoresed and then screened on 1.5% agarose gels, after which they were recovered using an EZNA^®^ Gel Extraction Kit (Omega, USA) in accordance with the manufacturer’s instructions.

A vector was connected to the PCR amplification products using a Zero Background pTOPO-Blunt Simple Cloning Kit (Aidlab Biotechnologies Co., Ltd) in accordance with the manufacturer’s instructions. For the selection of positive clones, 100 ng/μL ampicillin and universal M13 primers were used, and the positive clones were sent to Tshingke Biological Technology Co. (Wuhan, China) for sequencing.

The nucleotide sequences were aligned using MultAlin (http://multalin.toulouse.inra.fr/multalin/multalin.html). The amino acid sequences were deduced using Primer Premier 5, after which sequence alignment was carried out by ClustalW online (http://www.genome.jp/tools-bin/clustalw) and GeneDoc software.

#### Phylogenetic analysis

Homologous gene sequences of each carotenogenesis gene were obtained from the NCBI database (https://blast.ncbi.nlm.nih.gov/Blast.cgi). Their phylogenetic relationships were analyzed using MEGA 7.0.14. The maximum likelihood method was used to construct phylogenetic trees with 1000 replicate bootstrap supports, and values greater than 50% are shown at nodes.

### Data analysis

The developmental stages of ‘CH’ and ‘FC’ (Fig 2) were processed via Photoshop software. The metabolite and gene expression profiles were processed by OriginPro 8. Average significant difference analyses were examined at a threshold of *P*<0.05 and were conducted with ANOVA in SAS (SAS Institute, Inc., USA).

## Results

### Carotenoid production in the tissues of ‘CH’ and ‘FC’ pummelo fruits during development

‘CH’ and ‘FC’ pummelo fruits at five developmental stages were selected for analyses. The red-colored pigmentation in ‘CH’ started from the albedo at 75 DPA, followed by the SMs and JS at 100 DPA; this pigmentation ultimately peaked at 150 DPA in the SMs and JS. No red color was observed in any tissue of ‘FC’ at any stage (Fig 2). Combined with the content and composition of individual carotenoids detected by HPLC, lycopene contributed mainly to the red color in the albedo, SMs and JS of ‘CH’.

**Fig 3:**
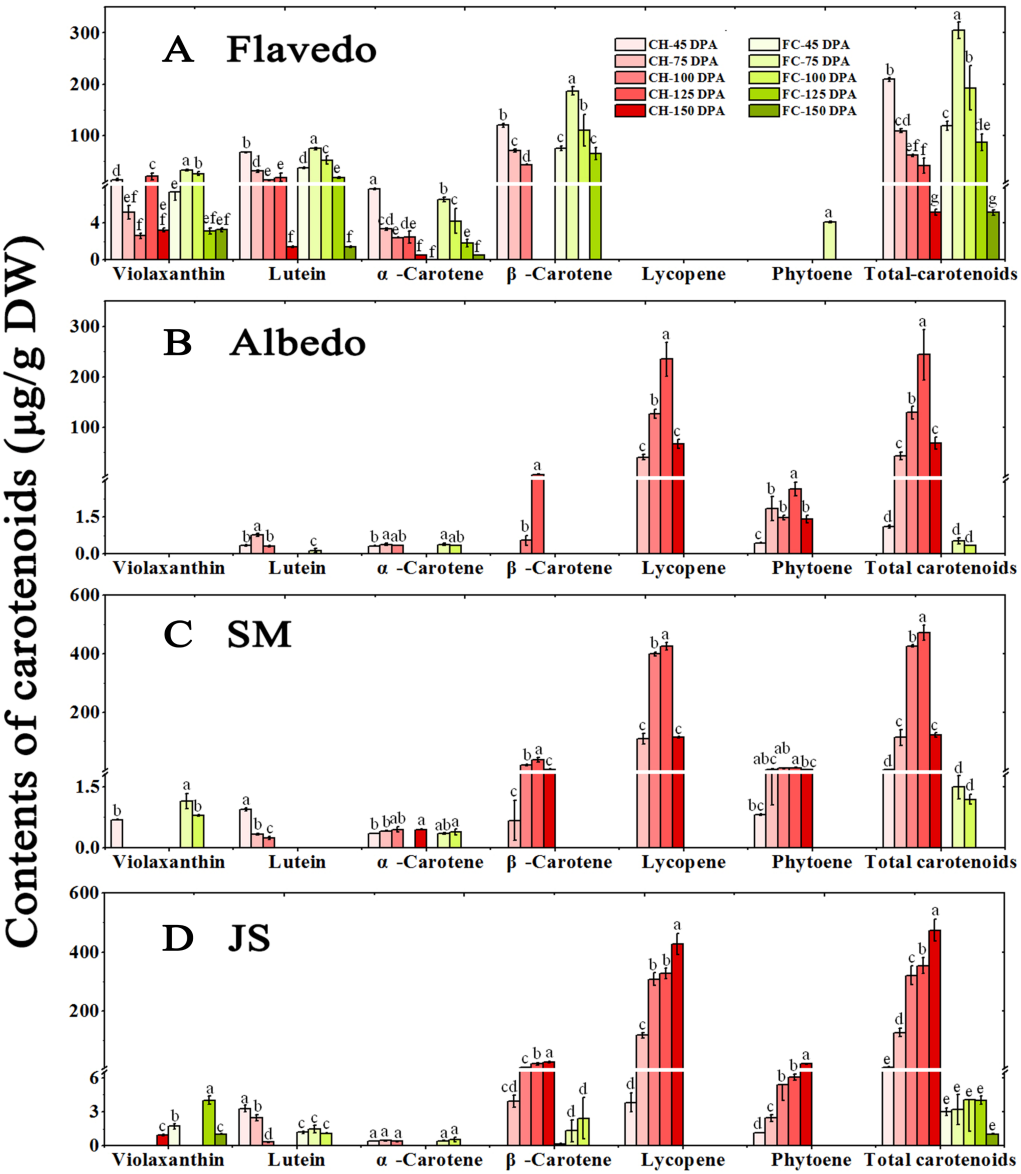
Carotenoid profiles in the tissues of two pummelo fruits during development (μg/g DW). Note: Columns represent the means (μg/g DW, n = 3) of the profiles of each carotenoid in the flavedo (A), albedo (B), SMs (segment membranes; C) and JS (juice sacs; D) in ‘CH’ and ‘FC’. a, b, c, etc. above each column indicate significant differences in carotenoid contents at the *P*<0.05 level among 5 stages of two pummelo fruits. DPA: days postanthesis. DW: dry weight.

In the flavedo, lutein, violaxanthin, α-carotene and β-carotene were found in both pummelo fruits during development. Except at the first stage, the total carotenoid content in ‘FC’ was significantly higher than that in ‘CH’, which is in accordance with our previous report concerning fruits at the commercially mature stage [11]. During fruit development, the total carotenoid content in ‘CH’ and ‘FC’ sharp decreased from 304.90 μg/g (dry weight) to 5.12 μg/g (dry weight). The dominant carotenoid in the flavedo was β-carotene, followed by lutein, before 125 DPA (Fig 3A).

In the albedo tissue, after the first stage (45 DPA), lycopene was the dominant carotenoid in ‘CH’, followed by phytoene and β-carotene. A dramatic accumulation of lycopene content in ‘CH’ was observed: it increased from trace levels at 45 DPA to the highest level at 125 DPA, after which it decreased beginning at 150 DPA. In contrast, a small amount of carotenoids were synthesized in ‘FC’. Only low levels of lutein at 75 DPA and α-carotene at both 75 DPA and 100 DPA were observed; all other carotenoids were at trace or nondetectable levels throughout the fruit development of ‘FC’ (Fig 3B).

In the SMs, the production of total carotenoids rapidly increased in ‘CH’ after 75 DPA. A large amount of lycopene accumulated, followed by β-carotene and phytoene. A dramatic accumulation of lycopene in ‘CH’ was observed from 45 DPA to 75 DPA. Afterward, the content increased by nearly 4-fold at 100 DPA and peaked at 125 DPA (accounting for 90.2% of the total carotenoids). However, lycopene and the total carotenoids both decreased dramatically at full maturity (150 DPA) (accounting for 93.9% of the total carotenoids). The contents of β-carotene and phytoene increased, exhibiting a trend similar to that of lycopene. In addition, the two carotenoids accounted for 6.2% of the total carotenoids after 45 DPA. However, low carotenoid biosynthesis was observed in ‘FC’, with only small amounts of violaxanthin and α-carotene detected at 75 DPA and 100 DPA, respectively (Fig 3C).

Likewise, in the JS of the ‘CH’ pummelo fruits, a large amount of lycopene was observed, followed by β-carotene and phytoene. A dramatic accumulation of lycopene in ‘CH’ was observed from 45 DPA to 75 DPA. Afterward, the level increased by nearly 3-fold at 100 DPA and peaked at 150 DPA, accounting for 89.5% of the total carotenoids. The contents of β-carotene and phytoene increased and exhibited a continuously increasing trend until the last development stage as did lycopene. On average, the two carotenoids accounted for 6.5% of the total carotenoids after 45 DPA, which is quite similar to the results in the SMs. As in the SMs, small amounts of carotenoids accumulated in the JS of ‘FC’; the highest levels were detected for violaxanthin at 125 DPA. Moreover, less contents of lutein, α-carotene and β-carotene were observed before 100 DPA, after which point the contents decreased to nondetectable or trace levels. In addition, undetectable or trace levels of phytoene and lycopene were observed at all stages of ‘FC’ (Fig 3D).

### Contents of limonin and nomilin in ‘CH’ and ‘FC’ pummelo fruits

**Fig 4:**
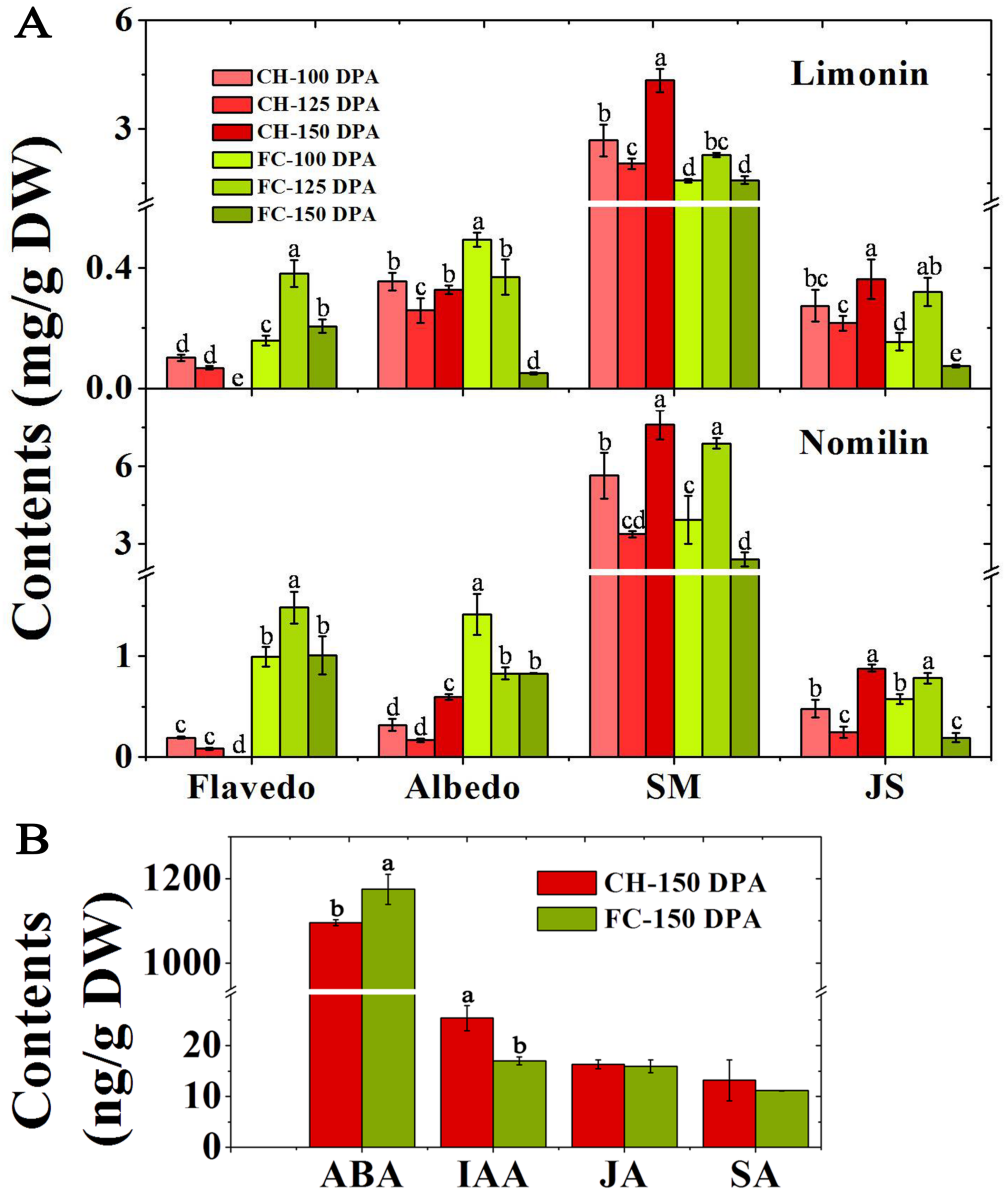
Limonin and nomilin contents (mg/g DW) in two pummelo fruits during development (A) as well as phytohormone contents (ng/g DW) at 150 DPA in two pummelo fruits (B). Note: A, the data shown are the means±standard deviations (SDs) (n = 3). * indicates a significance level of *P*<0.05 between two pummelo fruits. B, the data shown are the means±SEs (n = 3). ABA: abscisic acid. DPA: days postanthesis. IAA: indole-3-acetic acid; JA: jasmonic acid; SA: salicylic acid. a, b above the columns indicate significant differences at the *P*<0.05 level. SM: segment membrane. JS: juice sacs.

The contents of limonin and nomilin in the flavedo of ‘CH’ fruits were significantly lower than those in the ‘FC’ fruits from 100 DPA to 150 DPA. However, in the albedo, the contents of both limonoid aglycones in ‘CH’ were significantly lower than those in ‘FC’ at the two early stages, after which the contents dramatically increased at the last stage. However, both limonin and nomilin in the albedo of ‘FC’ continued to decrease to the lowest observed levels at 150 DPA. Interestingly, the highest limonin and nomilin production was observed in the SMs of both pummelo fruits; the limonin and nomilin contents in the SMs were 25.0-, 7.5-, 9.5-fold and 29.5-, 12.5-, 8.5-fold greater than those in the flavedo, albedo and JS of ‘CH’, respectively, and 5.5-, 3.0-, 7.0-fold and 2.0-, 2.5-, 6.5-fold greater than those in the flavedo, albedo and JS of ‘FC’, respectively. Notably, in SMs and JS, the limonin content was significantly higher in ‘CH’ than in ‘FC’ at both 100 DPA and 150 DPA. Additionally, at 150 DPA, the nomilin content in ‘CH’ was markedly higher than that in ‘FC’. In contrast, both the limonin and nomilin contents were significantly lower in ‘CH’ than in ‘FC’ at 125 DPA (Fig 4A).

### Phytohormone contents in ‘CH’ and ‘FC’ pummelo fruits

Owing to the large differences in the contents of carotenoids and limonoid aglycones in the JS at the last developmental stage, we measured the concentrations of phytohormones in the JS to determine whether such differences were caused by the regulation of phytohormones. ABA was lower in ‘CH’ than in ‘FC’, which was consistent with the results for Star Ruby (red-fleshed) and Marsh (white-fleshed) grapefruits [21], while IAA was higher in ‘CH’ than in ‘FC’. In addition, the contents of JA and SA were at the same levels in both pummelo fruits (Fig 4B).

#### Expression levels of carotenogenesis genes during fruit development

**Fig 5:**
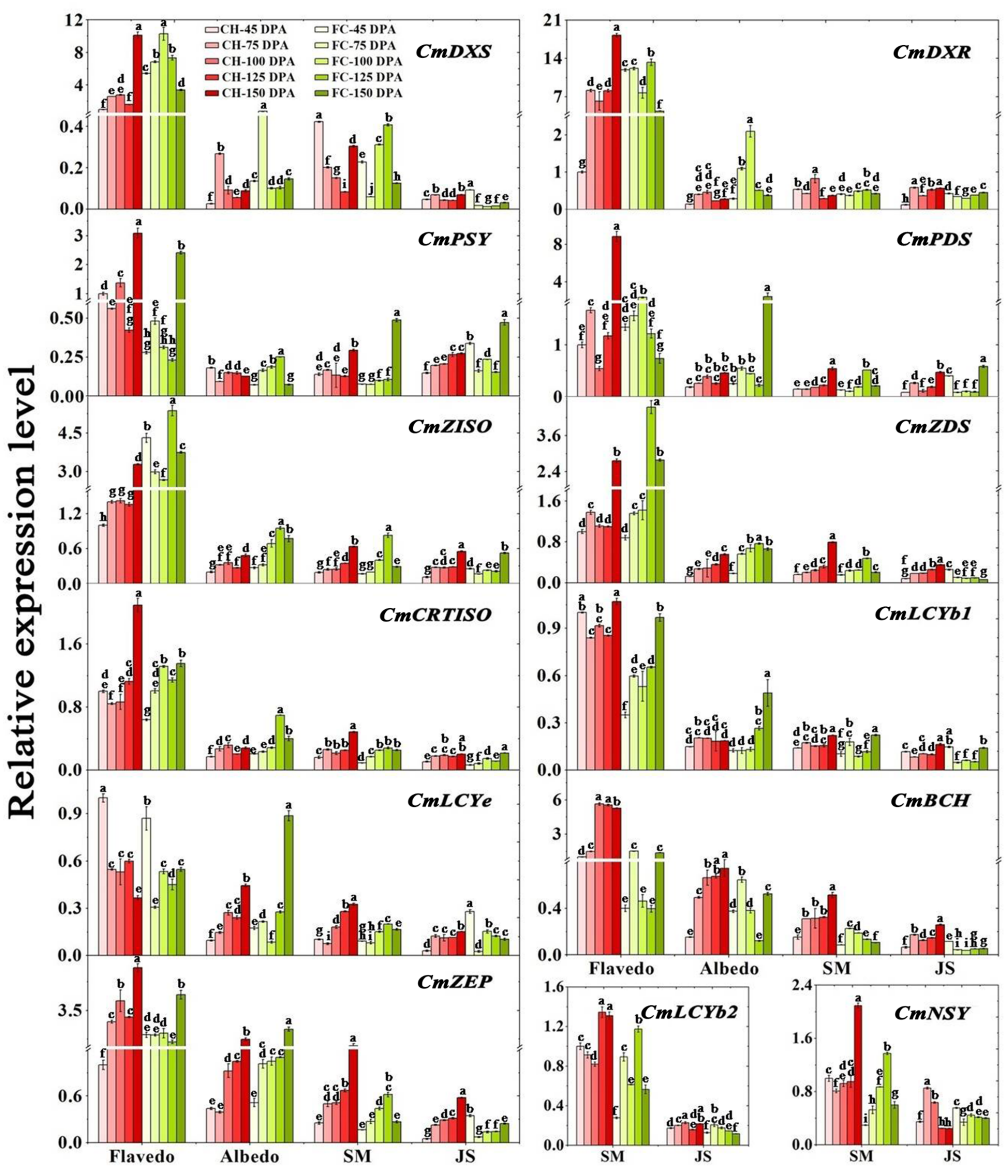
Carotenogenesis gene expression profiles in four tissues of ‘Chuhong’ and ‘Feicui’ pummelo fruits during different developmental stages. Note: The data shown are the means±standard deviations (SDs) (n = 3). a, b, c, etc. above each column indicate significant differences at the *P*<0.05 level. SM: segment membrane. JS: juice sacs.

In the flavedo, the expression levels of *CmDXS*, *CmDXR* and *CmZISO* were significantly higher in ‘FC’ than in ‘CH’ before 125 DPA, while the expression levels of *CmPSY*, lycopene β-cyclase gene 1 (*CmLCYb1)*, *CmBCH* and *CmZEP* were constantly higher in ‘CH’ throughout all developmental stages or during most developmental stages. At 150 DPA, the expression levels of only *CmZISO* and *CmLCYe* were downregulated in ‘CH’, and the expression level of only *CmZDS* was the same in both ‘CH’ and ‘FC’, whereas the expression levels of the other 8 genes were significantly upregulated in ‘CH’. Throughout all the stages, the largest fold change in gene expression was observed for *CmBCH*, followed by *CmPSY*, *CmPDS* and *CmZDS*, while the expression of *CmLCYb1* remained relatively stable (Fig 5).

In the albedo, the expression levels of *CmDXS*, *CmDXR*, *CmPDS*, *CmZDS*, *CmZISO*, *CmCRTISO* and *CmZEP* were significantly higher in ‘FC’ than in ‘CH’ (if not at the same level). However, compared with those in ‘CH’, the expression levels of *CmPSY*, *CmLCYb1* and *CmLCYe* in ‘FC’ were higher or the same at three stages. Notably, a generally increasing trend was observed in the expression levels of *CmPDS*, *CmZDS*, *CmZISO*, *CmLCYe* and *CmZEP* in both pummelo fruits, although larger fold changes occurred in ‘FC’. Compared with that in ‘FC’, the expression level of *CmLCYb1* in ‘CH’ was quite stable, but it drastically increased after 125 DPA (Fig 5).

The expression levels of 13 genes in the SMs were also evaluated. Higher expression levels of *CmZDS*, *CmLCYb1*, chromoplast-specific lycopene β-cyclase gene 2 (*CmLCYB2*), *CmLCYe*, *CmBCH* and *CmZEP* were detected in ‘CH’ than in ‘FC’ (if not at the same level). The expression levels of *CmDXS*, *CmDXR*, *CmPSY*, *CmPDS*, *CmZISO* and *CmCRTISO* were higher in ‘CH’ than in ‘FC’ in at least three stages. Notably, throughout all stages, the expression levels of *CmLCYe*, *CmBCH* and *CmZEP* were significantly higher in ‘CH’ than in ‘FC’. Interestingly, with the exceptions of the upregulation of *CmDXR*, *CmPSY* and *CmLCYb1*, the expression levels of the other nine genes were downregulated at 150 DPA in ‘FC’. Compared with that of the other genes, the expression of *CmLCYb1* in ‘CH’ was relatively steady. The expression levels of *CmLCYb2* and *CmNSY* were lower in ‘FC’ than in ‘CH’ at four of the five stages, while in ‘CH’ alone, the levels remained comparatively stable but were elevated at 125 DPA or 150 DPA (Fig 5).

In the JS, the expression levels of 13 genes were also determined. Notably, at 45 DPA, the expression levels of all the genes except *CmCRTISO* were higher in ‘FC’ than in ‘CH’. After 45 DPA, the expression levels of *CmZDS*, *CmBCH* and *CmZEP* were all significantly higher in ‘CH’ than in ‘FC’ (if not at the same level); the same results were obtained for *CmDXS*, *CmDXR*, *CmZDS*, *CmZISO* and *CmLCYb1*. Upregulated expression levels of *CmPSY*, *CmPDS* and *CmCRTISO* were observed at the last stage in ‘FC’. However, relatively steady expression levels of *CmDXS*, *CmLCYb1* and *CmLCYe* were observed in ‘CH’ throughout the fruit developmental stages (Fig 5).

### Alleles of carotenogenesis genes in red- and pale-/pale green-fleshed pummelo fruits

Considering the large amounts of carotenoids (especially lycopene) in the albedo, SMs and JS of ‘CH’, the sequences of 11 carotenogenesis genes were therefore checked for possible mutations. *CmPDS*, *CmZISO*, *CmBCH* and *CmNSY* shared one common allele in the JS of both pummelo fruits, while for the other 7 genes, two alleles were obtained for each gene (Table 1). The differences in nucleotide sequences of each carotenogenesis gene are shown in Table 1. Sites of various InDels and single amino acid polymorphisms are also indicated (S1–S11 Figs). Regarding *CmPSY*, which encodes the rate-limiting enzyme involved in carotenogenesis, *CmPSYa* and *CmPSYb*, which have one nucleotide InDel (site 183–185 causing CmPSYa^N^ and CmPSYb^-^ amino acid residues, respectively) and one different nonsynonymous site (site 215 causing CmPSYa^R^ and CmPSYb^Q^ amino acid residues, respectively), are present in ‘CH’ and ‘FC’, respectively (Table 1). However, in the other eight investigated pummelo fruits, the same differences at both sites of *CmPSY* were observed regardless of flesh color, and six alleles (referred to as CmPSYa to CmPSYf) were investigated (Table 1; S2 Fig). Thus, the difference in both sites might not be the factor that contributes to the large accumulation of carotenoids. However, among the four genes encoding enzymes that have cyclization functions in ‘CH’ and ‘FC’, the *CmNSY* alleles were identical in both pummelo fruits (Table 1), whereas for *CmLCYb1*, *CmLCYb2* and *CmLCYe*, two alleles were obtained for each gene (Table 1; S6–S8 Figs).

**Table 1:** Alleles of carotenogenesis genes in different tissues of ‘Chuhong’ and ‘Feicui’ pummelo (*Citrus maxima*) fruits.

Genes	Alleles ^(pummelos)^	Length of coding DNA sequence (CDS) bp (amino acid)	Different sites of nucleotides (amino acids) ^(site bp)^				
*CmDXR*	*CmDXRa* ^CH^	1422(473)	T(F) ^20(7)^	G(A) ^430(145)^			
	*CmDXRb* ^FC^	1422(473)	C(S) ^20(7)^	T(S) ^430(145)^			
*CmPSY*	*CmPSYa* ^CH, HR, HB, WS, FH, KP, T^	1317(438)	T(V) ^74(25)^	A(N) ^178(60)^	TAA(N) ^183-185(61)^	G(R) ^644(215)^	--
	*CmPSYb* ^HR, QDC, WBH, FC, FH^	1314(437)	T(V) ^74(25)^	A(N) ^178(60)^	--^183-185(61)^	A(Q) ^644(215)^	--
	*CmPSYc* ^HR, WBH, KP^	1314(438)	T(V) ^74(25)^	A(N) ^178(60)^	--^183-185(61)^	G(R) ^644(215)^	--
	*CmPSYd* ^QDC^	1314(437)	G(G) ^74(25)^	A(N) ^178(60)^	--^183-185(61)^	A(Q) ^644(215)^	--
	*CmPSYe* ^HB, WS, T^	1314(438)	T(V)^7 4(25)^	G(D) ^178(60)^	--^183-185(61)^	G(R) ^644(215)^	--
	*CmPSYf* ^WBH, FH, KP^	1402(397)	T(V) ^74(25)^	A(N) ^178(60)^	TAA(N) ^183-185(61)^	G(R) ^644(215)^	1147-1231 (85-bp base insertion; causing premature termination in amino acids, see S2A Fig)	
*CmPDS*	*CmPDS* ^CH, FC^	1659(552)					
*CmZISO*	*CmZISO* ^CH, FC^	1125(374)					
*CmCRTISO*	*CmCRTISOa* ^CH^	2028(675)	C(L) ^1360(454)^	G(M) ^1797(599)^			
	*CmCRTISOb* ^FC^	2028(675)	A(I) ^1360(454)^	A(I) ^1797(599)^			
*CmLCYb1*	*CmLCYb1a* ^CH, FC^	1515(504)	G(G) ^323(108)^				
	*CmLCYb1b* ^FC^	1515(504)	A(E) ^323(108)^				
*CmLCYb2*	*CmLCYb2a* ^CH, FC^	1512(503)	C(S) ^968(323)^				
	*CmLCYb2b* ^FC^	1512(503)	T(L) ^968(323)^				
*CmLCYe*	*CmLCYea* ^CH, FC^	1590(529)	A(I) ^307(103)^				
	*CmLCYeb* ^CH, FC^	1590(529)	T(F) ^307(103)^				
*CmBCH*	*CmBCH* ^CH, FC^	936(309)					
*CmZEP*	*CmZEPa* ^CH^	1995(664)	C(H) ^916(306)^	T(L) ^937(313)^	A(D) ^944(315)^	G(R) ^1538(513)^	T(V) ^1647(549)^
	*CmZEPb* ^FC^	1995(664)	A(N) ^916(306)^	G(V) ^937(313)^	G(G) ^944(315)^	A(Q) ^1538(513)^	G(G) ^1647(549)^
*CmNSY*	*CmNSY* ^CH, FC^	747(248)					

Note: The numbers and letters under ‘Different sites of nucleotides (amino acid) ^(site bp)^’ means the number of sites and different nucleotides (amino acids) among the alleles of each gene. --: deletion/no insertion. All single nucleotide polymorphisms shown above are nonsynonymous different sites. At least 10 clones for each carotenogenesis gene were sequenced from the cDNA mainly from the JS of each pummelo fruit. In particular, for *CmPSY*, *CmLCYb1*, *CmLCYb2*, *CmLCYe* and *CmNSY*, nearly 50 clones of each gene in each pummelo fruit were obtained. *CmDXR*: 1-deoxy-D-xylulose-5-phosphate reductoisomerase gene. *CmPSY*: phytoene synthase gene. *CmPDS*: phytoene desaturase gene. *CmCRTISO*: carotenoid isomerase gene. *CmZISO*: 15-*cis*-ζ-carotene isomerase gene. *CmLCYb1*: lycopene β-cyclase gene 1. *CmLCYb2*: lycopene β-cyclase gene 2. *CmLCYe*: lycopene ε-cyclase gene. *CmBCH*: β-carotenoid hydroxylase gene. *CmZEP*: zeaxanthin epoxidase gene. *CmNSY*: neoxanthin synthase gene. CH: ‘Chuhong’ pummelo (red-fleshed). FC: ‘Feicui’ pummelo (pale green-fleshed). HR: Huanong Red pummelo (red-fleshed). QDC: Chandler pummelo (red-fleshed). HB: Hirado Buntan pummelo (pink-fleshed). WBH: Wubu Red-fleshed pummelo (red-fleshed). T: Thai pummelo (pale yellow-fleshed). KP: Kao Pan (pale green-fleshed). FH: Fenghuang pummelo (pale green-fleshed). WS: Acidless pummelo (pale green-fleshed).

Moreover, a phylogenetic tree consisting of 11 genes was generated and analyzed via the full-length region of the studied genes and homologous genes obtained from the NCBI database (https://blast.ncbi.nlm.nih.gov/Blast.cgi); we named them the same as the closest previously reported genes in phylogenetic trees. The *CmDXR* and *CmZISO* genes were first cloned in citrus regardless of genome sequencing (S1–S11 Figs).

## Discussion

### Tissue-specific carotenogenesis in ‘CH’ and ‘FC’ pummelo fruits

Carotenoids are extremely important for plant growth and human dietary needs. Understanding the mechanism of carotenoid biosynthesis and accumulation is of great significance. In this study, we investigated the carotenoid profiles of two pummelo fruits that have different flesh colors. In ‘FC’, trace or small amounts of carotenoids were synthesized in the albedo, SMs and JS, while the accumulation of carotenoids was high in the flavedo. In accordance with the results of our previous report [11], high amounts of β-carotene, lutein and violaxanthin were detected in the flavedo of both pummelo fruits, and no lycopene was detected. Additionally, in the flavedo, except at 45 DPA, the production of total carotenoids was higher in ‘FC’ than in ‘CH’ at the corresponding stages.

However, in the albedo, SMs and JS of ‘CH’, the increased carotenogenesis could be largely attributed to the accumulation of lycopene, followed by that of β-carotene and phytoene. Thus, the data imply that the production of phytoene increased and that lycopene cyclization and β-carotene hydroxylation are blocked in the ‘CH’ fruit tissues. Moreover, the results of this study indicated that carotenoid synthesis is tissue specific, which is clearly demonstrated by the carotenoid synthesis in the flavedo and JS (Fig 3A and 3B). Clearly, different carotenoid profiles in the tissues of the two pummelo fruits indicate that the carotenoid biosynthesis pathway is relatively triggered in the JS of ‘CH’, while the pathway is suppressed in the flavedo during developmental stages. Moreover, the SMs of ‘CH’ also accumulated large amount of lycopene and thus more total carotenoids as observed in the JS; however, the reduction in these compounds in the SMs and the increase in the JS at the last stage again imply that carotenoid biosynthesis or lycopene accumulation is tissue specific, which is in good agreement with other reports in citrus fruit [15, 40] and carrot [41, 42].

### Changes in other metabolites in ‘CH’ and ‘FC’ pummelo fruits

In addition to the different carotenoid profiles in both pummelo fruits, differences in the production of other terpenoids such as volatile terpenoids have also been previously reported [5], and different contents of limonoid aglycones and phytohormones were also observed in the present study (Fig 4). Carotenoids, volatile terpenoids, limonoids and ABA are all terpenoids and share the same precursor pyruvate [43] (Fig 1), and the relationships among terpenoid metabolic pathways are complicated [27, 30, 44]. Moreover, ABA is a derivative of carotenoids and regulates carotenoid biosynthesis via feedback inhibition [17]. In the present study, lower ABA contents were detected in ‘CH’ than in ‘FC’, which was similar to results reported for Star Ruby (red-fleshed) and Marsh (white-fleshed) grapefruit [21], suggesting that carotenoid metabolism was blocked in ‘CH’. Ethylene is highly important for fruit ripening and influences the regulation of carotenoid production throughout fruit development. As a closely related phytohormone of ethylene, IAA also indirectly restricts the accumulation of carotenoids and thus plays a role in controlling the onset of fruit ripening [18]. In contrast, the endogenous IAA content was reduced in carotenoid-deficient tomato lines [45]. Compared with ‘FC’, ‘CH’ accumulated a higher IAA content at 150 DPA (Fig 4B), which also indicates an identical accumulation trend between carotenoids and IAA at the last stage. JA has been proposed to enhance lycopene accumulation in tomato fruits [46], and SA is a relatively far downstream product of β-carotene [47]. Thus, from the perspective of metabolomic networks, in red-fleshed ‘CH’, other terpenoids are involved in the accumulation mechanism of carotenes, including phytoene, lycopene and β-carotene. However, it cannot be presumed that the largest difference in metabolism between ‘CH’ and ‘FC’ lies in the carotenoid biosynthesis pathway until their entire metabolic profiles are revealed.

### Critical genes involved in the carotenoid biosynthesis pathway

Based on our results of carotenogenesis, it might not be appropriate to simply attribute the accumulation of lycopene, phytoene or β-carotene in ‘CH’ to the upregulated expression of upstream genes and the downregulated expression of downstream genes, as Liu *et al*. [22] reported for sweet oranges. It would be very helpful if the functions of the enzymes coded by various alleles of all related genes were clarified, which will require additional reliable and rigid verification due to the large numbers of alleles of all the investigated genes. In this study, the expression of two genes in the MEP pathway upstream of the carotenoid biosynthetic pathway and eleven genes in the latter pathway, as well as the alleles of the eleven genes, was evaluated (Fig 5; Table 1). Overall, the higher expression of *CmZDS*, *CmBCH* and *CmZEP* might contribute to the higher lycopene content in the SMs and JS of ‘CH’. Except at the first stage, the expression levels of genes involved in the MEP pathway (*CmDXR* and *CmDXS*) were significantly higher in the JS of ‘CH’ than in those of ‘FC’, implying that the biosynthesis of the initial carotenoid metabolites in the carotenogenesis pathway was largely induced in ‘CH’. With respect to the rate-limiting gene *CmPSY* in carotenogenesis, we observed differences in two sites between *CmPSYa* and *CmPSYb* (Table 1). Considering that ‘AAT’ repeats are correlated with high carotenoid (especially lycopene and β-carotene) accumulations in citrus [48], we rechecked for ‘AAT’ repeats (‘N’ repeats at the amino acid level) and carotenoid accumulation in the 10 pummelo fruits investigated (Table 1). Both *CmPSY* alleles are present in pummelo fruits that present flesh of various colors (Table 1), suggesting that the differences in *CmPSY* alleles are not associated with flesh color. Posttranslational regulation is important with respect to supporting the function of carotenogenesis enzymes. Two alternative splice variants with different 5' untranslated regions (UTRs) were identified in the *Arabidopsis* PSY gene; these variants resulted in different translation rates for regulating AtPSY enzyme activity [49].

The *LCYb1* [31, 32, 44, 50], *LCYb2* [41, 42, 44, 50, 51], *LCYe* [50] and *NSY* [52] genes function in catalyzing the cyclization of lycopene. Compared with the nonfunctional β-LCY2b, functional *β-LCY2a* was shown to have higher expression levels in navel oranges and white-fleshed marsh grapefruits, whereas nonfunctional *β-LCY2b* was expressed at much higher levels in red-fleshed Star Ruby fruits [21, 31]. Since β-carotene (the direct product of β-LCY) also significantly accumulates in Star Ruby fruit [19, 21, 31], it cannot be excluded that functional LCYb and other cyclases such as LCYe and NSY are responsible for the accumulation of β-carotene [32, 52]. In addition, the higher expression levels of *CmLCYb1* and *CmLCYb2* in the JS of ‘CH’ might explain the higher β-carotene content throughout the fruit development stages.

Notably, the expression of the downstream genes *CmBCH* and *CmZEP* was constantly significantly higher in the JS of ‘CH’ than in those of ‘FC’, which is similar to results reported in Star Ruby grapefruit [31] but opposite those in ‘Cara cara’ navel orange [13]. Yan *et al*. reported that maize *crtRB1* alleles that encode an enzyme that exhibits BCH function are associated with reduced transcript expression and higher β-carotene concentrations [53]. However, in the present study, both the expression levels of *CmBCH* and the β-carotene accumulation were higher in the albedo, SMs and JS of ‘CH’ than in those of ‘FC’ at most development stages (Figs 3 and 5). Interestingly, compared with wild-type *Arabidopsis* plants, *Arabidopsis* mutants lacking ZEP activity exhibited a marked sixfold increase in total seed carotenoid contents, indicating that *ZEP* is a major contributor to carotenoid contents [54]. Furthermore, fruit-specific RNAi-mediated suppression of the 9-cis-epoxycarotenoid dioxygenase (*SlNCED1*) gene was accompanied by increased contents of lycopene and β-carotene in tomato fruits [55]. The carotenoid biosynthesis, maintenance and degradation genes also turned out to be regulatory factors involved in *Actinidia* fruit development [56].

### Other factors that might regulate carotenoid biosynthesis

Transcription factors also play important roles in carotenoid accumulation. Overexpression of CsMADS6 resulted in higher lycopene contents and higher β-carotene contents in the sepals and fruits of tomato, respectively, by binding the promoters of *LCYb1*, *PSY*, *PDS* and *CCD1* and upregulating their expression [57]. In a stay-green Ougan mutant (*Citrus reticulata*), CrMYB68 downregulated the expression of two carotenoid pathway genes, *CrBCH* and *CrNCED5*, which resulted in total flavedo carotenoid contents that were lower than those in the wild type [17]. The *Mimulus lewisii* R2R3 MYB transcription factor mutant *rcp1* contains low amounts of carotenoids exhibits low expression levels of almost all carotenoid biosynthesis pathway genes [58]. Luo *et al*. reported that a stay-green protein, SlSGR1, dramatically reduced lycopene biosynthesis via the direct inhibition of SlPSY1 activity [59]. However, only one of these factors, ripening inhibitor (RIN), has been confirmed to regulate carotenoid accumulation in tomato fruits via interaction with the promoter of *SlPSY1* [60]. Phytochrome-interacting factors (PIFs) bind to the *PSY* promoter, which suppresses its expression and reduces carotenoid accumulation in *Arabidopsis* [61]. Overall, since we did not find critical variants in carotenogenesis genes, the upstream regulators deserve extensive study in future research.

## Conclusion

The development stage in which dramatic carotenoid production in the albedo, SMs and JS of ‘CH’ occurred was from 45 DPA to 75 DPA, and the total carotenoids increased by more than 39-, 40- and 14-fold, respectively. Different expression levels of carotenogenesis genes, especially *CmZDS*, *CmBCH* and *CmZEP*, in combination might be directly responsible for the largely different carotenoid profiles between these two pummelo fruits. The lycopene accumulation in specific ‘CH’ fruit tissues might strongly be caused by the different alleles of the *CmDXR*, *CmPSY*, *CmCRTISO*, *CmLCYb1*, *CmLCYb2*, *CmLCYe*, and *CmZEP* genes. The different numbers of ‘AAT’ microsatellite repeat units in *PSY* transcripts were not related to lycopene accumulation in red-flesh pummelo fruits.

## Supporting information

S1 Fig Sequence analysis of CmDXR in ‘CH’ and ‘FC’. A: CmDXRa and CmDXRb were detected in ‘CH’ and ‘FC’, respectively. Two amino acid differences in sequences were observed between CmDXRa and CmDXRb. B: Phylogenetic analysis of CmDXR.
(DOC)

S2 Fig Sequence analysis of CmPSY in ten pummelos. A: Six CmPSY alleles were detected in ten pummelo fruits. The orange framework means ‘N’ repeats. B: Phylogenetic analysis of CmPSY.
(DOC)

S3 Fig Sequence analysis of CmPDS in ‘CH’ and ‘FC’. A: CmPDS was identical between ‘CH’ and ‘FC’. B: Phylogenetic analysis of CmPDS.
(DOC)

S4 Fig Sequence analysis of CmZISO in ‘CH’ and ‘FC’. A: CmZISO was identical between ‘CH’ and ‘FC’. B: Phylogenetic analysis of CmZISO.
(DOC)

S5 Fig Sequence analysis of CmCRTISO in ‘CH’ and ‘FC’. A: CmCRTISOa and CmCRTISOb were detected in ‘CH’ and ‘FC’, respectively. The red frameworks show different sites between CmCRTISOa and CmCRTISOb. Two amino acid differences in sequences were observed between ‘CH’ and ‘FC’. B: Phylogenetic analysis of CmCRTISO.
(DOC)

S6 Fig Sequence analysis of CmLCYb1 in ‘CH’ and ‘FC’. A: CmLCYb1a and CmLCYb1b were detected in ‘CH’ and ‘FC’, respectively. Note that single amino acid differences in sequences were observed between CmLCYb1a and CmLCYb1b. B: Phylogenetic analysis of CmLCYb1.
(DOC)

S7 Fig Sequence analysis of CmLCYb2 in ‘CH’ and ‘FC’. A: CmLCYb2a and CmLCYb2b were detected mainly in ‘CH’ and ‘FC’, respectively. Note that a single amino acid difference in sequences was observed between CmLCYb2a and CmLCYb2b. B: Phylogenetic analysis of CmLCYb2.
(DOC)

S8 Fig Sequence analysis of CmLCYe in ‘CH’ and ‘FC’. A: CmLCYea and CmLCYeb were detected in ‘CH’ and ‘FC’, respectively. A single amino acid difference in sequences was observed between CmLCYea and CmLCYeb. B: Phylogenetic analysis of CmLCYe.
(DOC)

S9 Fig Sequence analysis of CmBCH in ‘CH’ and ‘FC’. A: CmBCH was identical between ‘CH’ and ‘FC’. B: Phylogenetic analysis of CmBCH.
(DOC)

S10 Fig Sequence analysis of CmZEP in ‘CH’ and ‘FC’. A: CmZEPa and CmZEPb were detected in ‘CH’ and ‘FC’, respectively. A single amino acid difference in sequences was observed between CmZEPa and CmZEPb. B: Phylogenetic analysis of CmZEP.
(DOC)

S11 Fig Sequence analysis of CmNSY in ‘CH’ and ‘FC’. A: CmNSY was identical between ‘CH’ and ‘FC’. B: Phylogenetic analysis of CmNSY.
(DOC)

S1 Table Primers used for quantitative real-time PCR (qRT-PCR) analysis. The accession number was obtained from the *Citrus sinensis* (Valencia orange) annotation project (http://citrus.hzau.edu.cn/orange/).
(DOC)

S2 Table Primers used for gene cloning. The superscript ‘a’ gene accession means that the accession number was obtained from the NCBI database (http://www.ncbi.nlm.nih.gov/). The superscript ‘b’ genome accession means that the accession number was obtained from the *Citrus sinensis* (Valencia orange) annotation project (http://citrus.hzau.edu.cn/orange/).
(DOC)
