# Supplementary material for: Largely different carotenogenesis in two pummelo fruits with different flesh colors
Source: PLoS One. 2018 Jul 9;13(7):e0200320. doi: 10.1371/journal.pone.0200320 (PMC6037374; doi:10.1371/journal.pone.0200320)
Supplement: S1 Fig — A: CmDXRa and CmDXRb were detected in ‘CH’ and ‘FC’, respectively. Two amino acid differences in sequences were observed between CmDXRa and CmDXRb. B: Phylogenetic analysis of CmDXR. (DOC) [file pone.0200320.s001.doc]

A

B

*Prunus persica* DXR (XP_007210092.2)

*Prunus avium* DXR (XP_021826007.1)

*Pyrus communis* DXR (AIR72281.1)

*Eriobotrya japonica* DXR (AFP43697.1)

*Rosa hybrid cultivar* DXR (AFR79418.1)

*Rosa rugosa* DXR (AEZ53171.1)

*Arabidopsis lyrata* DXR (XP_002866510.1)

*Brassica napus* DXR (XP_013661183.1)

*Cephalotus follicularis* DXR (GAV63780.1)

*Arachis ipaensis* DXR (XP_016193847.1)

*Arachis duranensis* DXR (XP_015968127.1)

*Medicago truncatula* DXR (XP_003608963.2)

**CmDXRa**

**CmDXRb**

*Morus alba* DXR (AOV62774.1)

*Morus notabilis* DXR (XP_010101212.1)

100

100

100

100

100

98

99

94

97

96

56

0.02

**S1 Fig. Sequence analysis of CmDXR in 'CH' and 'FC'.**

Note: A: CmDXRa and CmDXRb were detected in 'CH' and 'FC', respectively. Two amino acid differences in sequences were observed between CmDXRa and CmDXRb. B: Phylogenetic analysis of CmDXR.
