# Supplementary material for: Largely different carotenogenesis in two pummelo fruits with different flesh colors
Source: PLoS One. 2018 Jul 9;13(7):e0200320. doi: 10.1371/journal.pone.0200320 (PMC6037374; doi:10.1371/journal.pone.0200320)
Supplement: S2 Fig — A: Six CmPSY alleles were detected in ten pummelo fruits. The orange framework means ‘N’ repeats. B: Phylogenetic analysis of CmPSY. (DOC) [file pone.0200320.s002.doc]

A

B

**CmPSYc**

*Citrus maxima* PSY (AJT59421.1)

**CmPSYa**

**CmPSYe**

**CmPSYf**

*Citrus* x *paradisi* PSY (AAD38051.2)

**CmPSYb**

**CmPSYd**

*Citrus* x *microcarpa* PSY (ABY86651.1)

*Mangifera indica* PSY (AFE85918.1)

*Bixa orellana* PSY (AMJ39471.1)

*Theobroma cacao* PSY (EOY03122.1)

*Jatropha curcas* PSY (XP_012074703.1)

*Hevea brasiliensis* PSY (XP_021645276.1)

*Manihot esculenta* PSY (XP_021592580.1)

*Momordica charantia* PSY (XP_022141680.1)

*Momordica cochinchinensis* PSY (AGW16352.1)

*Cucurbita pepo* PSY (AFV33361.1)

*Cucumis melo* PSY (NP_001284425.1)

*Citrullus lanatus* PSY (AGT57744.1)

*Luffa aegyptiaca* PSY (APO14283.1)

85

99

79

100

85

100

52

97

100

0.02

**S2 Fig. Sequence analysis of CmPSY in ten pummelo fruits.**

Note: A: Six CmPSY alleles were detected in ten pummelo fruits. The orange framework means 'N' repeats. B: Phylogenetic analysis of CmPSY.
