# Supplementary material for: Largely different carotenogenesis in two pummelo fruits with different flesh colors
Source: PLoS One. 2018 Jul 9;13(7):e0200320. doi: 10.1371/journal.pone.0200320 (PMC6037374; doi:10.1371/journal.pone.0200320)
Supplement: S3 Fig — A: CmPDS was identical between ‘CH’ and ‘FC’. B: Phylogenetic analysis of CmPDS. (DOC) [file pone.0200320.s003.doc]

A

>CmPDS

MSLCFSVSESAFNLRYGFRDSEPMGQSLKIRVKTRTRKGFCPSKVVCVDYPRPDIDNTSNFLEAAYLSSSFRTSPRPSKPLKVVIAGAGLAGLSTAKYLADAGHKPLLLEARDVLGGKIAAWKDGDGDWYETGLHIFFGAYPNIQNLFGELGINDRLQWKEHSMIFAMPNKPGEFSRFDFPEVLPAPLNGILAILRNNEMLTWPEKVKFAIGLLPAIIGGQAYVEAQDGLTVQEWMRKQGVPDRVTTEVFIAMSKALNFINPDELSMQCILIALNRFLQEKHGSKMAFLDGNPPERLCLPIVEHIQSLGGEVRLNSRVQKIELNDDGTVKNFLLTNGNVIDGDAYVFVTPVDILKLQLPENWKEMAYFKRLEKLVGVPVINIHIWFDRKLKNTYDHLLFSRSPLLSVYADMSLTCKEYYNPNQSMLELVFAPAEEWISCSDSEIIDATMKELAKLFPDEISADQSKAKIVKYHVVKTPRSVYKTIPNCEPCRPLQRSPVEGFYLAGDYTKQKYLASMEGAVLSGKLCAQAIVQDYVLLAARGKGRLAEASMC-

B

**CmPDS**

*Citrus* x *paradisi* PDS (AAK51545.1)

*Citrus maxima* PDS (AJT59422.1)

*Citrus sinensis* PDS (AEQ29518.1)

*Populus trichocarpa* PDS (XP 002321104.2)

*Hevea brasiliensis* PDS (XP 021691516.1)

*Manihot esculenta* PDS (XP 021613095.1)

*Prunus armeniaca* PDS (AAX33347.1)

*Malus domestica* PDS (ANS58097.1)

*Momordica charantia* PDS (XP 022159101.1)

*Cucurbita moschata* PDS (AEK86565.1)

*Cucumis melo* PDS (NP 001284459.1)

*Helianthus annuus* PDS (AHA36971.1)

*Chrysanthemum* x *morifolium* PDS (BAE79552.1)

*Camellia sinensis* PDS (AHB32104.1)

*Rhododendron molle* PDS (APB08594.1)

*Diospyros kaki* PDS (ACY78343.1)

*Macleaya cordata* PDS (OVA16057.1)

*Vitis vinifera* PDS (AFP28796.1)

*Sesamum indicum* PDS (AHV90407.1)

*Catharanthus roseus* PDS (AGH32499.1)

*Petunia* x *hybrida* PDS (AKM12416.1)

*Lycium chinense* PDS (AHN92038.1)

*Nicotiana benthamiana* PDS (ABE99707.1)

73

100

94

100

100

83

100

100

96

66

58

72

53

51

0.02

**S3 Fig.** **Sequence analysis of CmPDS in 'CH' and 'FC'.**

Note: A: CmPDS was identical between 'CH' and 'FC'. B: Phylogenetic analysis of CmPDS.
