# Supplementary material for: Largely different carotenogenesis in two pummelo fruits with different flesh colors
Source: PLoS One. 2018 Jul 9;13(7):e0200320. doi: 10.1371/journal.pone.0200320 (PMC6037374; doi:10.1371/journal.pone.0200320)
Supplement: S4 Fig — A: CmZISO was identical between ‘CH’ and ‘FC’. B: Phylogenetic analysis of CmZISO. (DOC) [file pone.0200320.s004.doc]

A

>CmZISO

MSSSSCLLLSSSVPRIKKEAFANIRTKRDASTSASVSISCKLKPAPPCPLTLFFNSNPAKQKIVLVRSRTETGSGTDSDTDLATLAGEDSAAFDLKNQKLTSWVYFSVILGVVLFLLQLLWIDNSTGYGKAFIDSVSSLSDSHEVVMLVLILIFATVHSGLASLRDMGEKVIGARAYRVLFAGVSLPLAVSTIVYFINHRYDGMQLWQLQGAPGVHQIVWLSSFVSFFFLYPSTFNLLEVAAVDEPKMHLWETGVMRITRHPQMVGQVIWCLAHTLWIGNSVAAAASLGLIGHHLFGVWNGDKRLATRYGEAFEAVKRRTSVIPFAAIITGRQILPKDYYKEFIRLPYLTITALTLGAYIAHPLMQSASFLLHW-

B

*Brassica napus* ZISO (XP 013645558.1)

*Arabidopsis thaliana* ZISO (NP 563879.1)

**CmZISO**

*Carica papaya* ZISO (XP 021907216.1)

*Spinacia oleracea* ZISO (XP 021861125.1)

*Chenopodium quinoa* ZISO (XP 021748968.1)

*Dendrobium catenatum* ZISO (XP 020690862.1)

*Crocus ancyrensis* ZISO (ALQ56934.1)

*Asparagus officinalis* ZISO (XP 020245806.1)

*Ananas comosus* ZISO (XP 020111958.1)

*Amborella trichopoda* ZISO (XP 006848466.1)

100

100

96

62

53

0.05

**S4 Fig. Sequence analysis of CmZISO in 'CH' and 'FC'.**

Note: A: CmZISO was identical between 'CH' and 'FC'. B: Phylogenetic analysis of CmZISO.
