# Supplementary material for: Largely different carotenogenesis in two pummelo fruits with different flesh colors
Source: PLoS One. 2018 Jul 9;13(7):e0200320. doi: 10.1371/journal.pone.0200320 (PMC6037374; doi:10.1371/journal.pone.0200320)
Supplement: S5 Fig — A: CmCRTISOa and CmCRTISOb were detected in ‘CH’ and ‘FC’, respectively. The red frameworks show different sites between CmCRTISOa and CmCRTISOb. Two amino acid differences in sequences were observed between ‘CH’ and ‘FC’. B: Phylogenetic analysis of CmCRTISO. (DOC) [file pone.0200320.s005.doc]

A

B

*Herrania umbratica* CRTISO (XP 021288983.1)

*Theobroma cacao* CRTISO (EOY08564.1)

*Corchorus capsularis* CRTISO (OMO95042.1)

*Corchorus olitorius* CRTISO (OMO68668.1)

*Bixa orellana* CRTISO (AMJ39478.1)

*Populus tomentosa* CRTISO (APX43201.1)

*Jatropha curcas* CRTISO (XP 012074260.1)

*Hevea brasiliensis* CRTISO (XP 021667983.1)

*Manihot esculenta* CRTISO (XP 021619584.1)

*Carica papaya* CRTISO (XP 021886924.1)

*Malus domestica* CRTISO (NP 001315958.1)

*Prunus persica* CRTISO (XP 020423714.1)

*Spinacia oleracea* CRTISO (XP 021858982.1)

*Macleaya cordata* CRTISO (OVA16223.1)

*Citrus sinensis* CRTISO (KDO70702.1)

**CmCRTISOa**

**CmCRTISOb**

89

100

100

99

100

100

86

83

89

51

50

0.02

**S5 Fig. Sequence analysis of CmCRTISO in 'CH' and 'FC'.**

Note: A: CmCRTISOa and CmCRTISOb were detected in 'CH' and 'FC', respectively. The red frameworks show different sites between CmCRTISOa and CmCRTISOb. Two amino acid differences in sequences were observed between 'CH' and 'FC'. B: Phylogenetic analysis of CmCRTISO.
