# Supplementary material for: Largely different carotenogenesis in two pummelo fruits with different flesh colors
Source: PLoS One. 2018 Jul 9;13(7):e0200320. doi: 10.1371/journal.pone.0200320 (PMC6037374; doi:10.1371/journal.pone.0200320)
Supplement: S6 Fig — A: CmLCYb1a and CmLCYb1b were detected in ‘CH’ and ‘FC’, respectively. Note that single amino acid differences in sequences were observed between CmLCYb1a and CmLCYb1b. B: Phylogenetic analysis of CmLCYb1. (DOC) [file pone.0200320.s006.doc]

A

B

*Citrus* x *paradisi* LCY(AEQ29514.1)

*Citrus* x *paradisi* LCYb (AEQ29517.1)

**CmLCYb1a**

**CmLCYb1b**

*Citrus sinensis* LCYb2 (ABF69942.1)

*Citrus sinensis* LCYb2 (ACP19704.1)

*Citrus maxima* LCYb (AAR89632.1)

*Citrus unshiu* LCYb (AAN86060.1)

*Citrus sinensis* LCYb1 (NP_001275849.1)

*Durio zibethinus* LCYb (ARH12814.1)

*Corchorus olitorius* LCYb (OMO60094.1)

*Theobroma cacao* LCYb (EOY09059.1)

*Herrania umbratica* LCYb (XP_021282724.1)

*Cephalotus follicularis* LCYb (GAV81497.1)

*Populus trichocarpa* LCYb (XP_002323260.1)

*Jatropha curcas* LCYb (XP_012085981.1)

*Manihot esculenta* LCYb (XP_021623586.1)

*Carica papaya* LCYb (ABD91578.1)

*Adonis aestivalis* LCYb (AAK07430.1)

97

100

90

66

93

78

100

65

0.02

**S6 Fig.** **Sequence analysis of CmLCYb1 in 'CH' and 'FC'.**

Note: A: CmLCYb1a and CmLCYb1b were detected in 'CH' and 'FC', respectively. Note that single amino acid differences in sequences were observed between CmLCYb1a and CmLCYb1b. B: Phylogenetic analysis of CmLCYb1.
