# Supplementary material for: Largely different carotenogenesis in two pummelo fruits with different flesh colors
Source: PLoS One. 2018 Jul 9;13(7):e0200320. doi: 10.1371/journal.pone.0200320 (PMC6037374; doi:10.1371/journal.pone.0200320)
Supplement: S7 Fig — A: CmLCYb2a and CmLCYb2b were detected mainly in ‘CH’ and ‘FC’, respectively. Note that a single amino acid difference in sequences was observed between CmLCYb2a and CmLCYb2b. B: Phylogenetic analysis of CmLCYb2. (DOC) [file pone.0200320.s007.doc]

A

B

**Cm LCYb2a**

*Citrus maxima* LCY2b (AJT59426.1)

**Cm LCYb2b**

*Citrus* x *paradisi* LCYb (ACX37456.1)

*Jatropha curcas* CCS (XP_012070159.1)

*Macleaya cordata* CYC (OVA10035.1)

*Vitis vinifera* CCS NP_001304061.1

*Cynara cardunculus* LCYb/e (KVH90663.1)

*Camellia nitidissima* NSY (ADZ28517.1)

*Actinidia chinensis* LCYb (ACJ66628.1)

*Actinidia deliciosa* LCYb (ACJ66629.1)

*Sesamum indicum* CCS (XP_011088917.1)

*Erythranthe lewisii* LCYb1 (ALE33751.1)

*Dorcoceras hygrometricum* LCY (KZV17368.1)

*Lycium barbarum* LCYb (AIX87499.1)

*Solanum lycopersicum* NSY (NP_001234445.2)

*Capsicum annuum* CCS (AIQ82719.1)

100

100

75

100

100

92

99

89

59

50

0.05

**S7 Fig. Sequence analysis of CmLCYb2 in 'CH' and 'FC'.**

Note: A: CmLCYb2a and CmLCYb2b were detected mainly in 'CH' and 'FC', respectively. Note that a single amino acid difference in sequences was observed between CmLCYb2a and CmLCYb2b. B: Phylogenetic analysis of CmLCYb2.
