# Supplementary material for: Largely different carotenogenesis in two pummelo fruits with different flesh colors
Source: PLoS One. 2018 Jul 9;13(7):e0200320. doi: 10.1371/journal.pone.0200320 (PMC6037374; doi:10.1371/journal.pone.0200320)
Supplement: S8 Fig — A: CmLCYea and CmLCYeb were detected in ‘CH’ and ‘FC’, respectively. A single amino acid difference in sequences was observed between CmLCYea and CmLCYeb. B: Phylogenetic analysis of CmLCYe. (DOC) [file pone.0200320.s008.doc]

A

B

*Theobroma cacao* LCYe (EOY30388.1)

*Herrania umbratica* LCYe (XP_021276527.1)

*Durio zibethinus* LCYe (XP_022751743.1)

*Abelmoschus esculentus* LCYe (APO14286.1)

*Jatropha curcas* LCYe (XP_012076949.1)

*Hevea brasiliensis* LCYe (XP_021637952.1)

*Manihot esculenta* LCYe (XP_021596942.1)

*Citrus sinensis* LCYe (AAS48096.1)

**CmLCYeb**

**CmLCYea**

*Citrus maxima* LCYe (AJT59425.1)

*Carica papaya* LCYe (XP_021902015.1)

*Elaeagnus umbellata* LCYe (AOH73308.1)

*Momordica charantia* LCYe (XP_022154629.1)

*Cucumis melo* LCYe (NP_001315403.1)

*Arachis ipaensis* LCYe (XP_016189142.1)

*Cajanus cajan* LCYe (XP_020220608.1)

*Medicago truncatula* LCYe (XP_003595243.1)

82

100

100

99

90

99

100

58

93

86

72

56

78

0.02

**S8 Fig. Sequence analysis of CmLCYe in 'CH' and 'FC'.**

Note: A: CmLCYea and CmLCYeb were detected in 'CH' and 'FC', respectively. A single amino acid difference in sequences was observed between CmLCYea and CmLCYeb. B: Phylogenetic analysis of CmLCYe.
