# Supplementary material for: Largely different carotenogenesis in two pummelo fruits with different flesh colors
Source: PLoS One. 2018 Jul 9;13(7):e0200320. doi: 10.1371/journal.pone.0200320 (PMC6037374; doi:10.1371/journal.pone.0200320)
Supplement: S9 Fig — A: CmBCH was identical between ‘CH’ and ‘FC’. B: Phylogenetic analysis of CmBCH. (DOC) [file pone.0200320.s009.doc]

A

>CmBCH

MAVGLLAAIVPKPFCLLTTKLQPSSLLTTKPAPLYAPLGTHHGFFNGKNRRKLNSFTVCFVLEEKKQSTQIETFTEEEEEESGTQISTAARVAEKLARKRSERFTYLVAAVMSSFGITSMAVMAVYYRFWWQMEGGEVPLAEMFGTFALSVGAAVGMEFWARWAHKALWHASLWHMHESHHRPREGPFELNDVFAIINAVPAIALLSFGFFHKGLVPGLCFGAGLGITVFGMAYMFVHDGLVHKRFPVGPIADVPYFRRVAAAHQLHHSDKFHGVPYGLFLGPKELEEVEGLEELEKEISKRIKSYNRVPK-

B

**CmBCH**

*Citrus kinokuni* BCH (CAL63739.1)

*Citrus unshiu* BCH (AAG33636.1)

*Citrus sinensis* BCH (NP_001275830.1)

*Phellodendron amurense* BCH (ATL77042.1)

*Prunus persica* BCH (XP_007218715.1)

*Vigna radiata* BCH (XP_014503371.1)

*Bixa orellana* BCH (AMJ39484.1)

*Theobroma cacao* BCH (EOY23306.1)

*Corchorus olitorius* BCH (OMP03964.1)

*Gentiana lutea* BCH (BAE92729.1)

*Sesamum indicum* BCH (XP_011084458.1)

*Coffea arabica* BCH (ABA43903.1)

*Lycium barbarum* BCH (AIX87502.1)

*Ipomoea nil* BCH (BAI47580.1)

*Ipomoea obscura* BCH (BAI47579.1)

*Momordica charantia* BCH (XP_022141331.1)

*Arabidopsis lyrata* BCH (XP_020873639.1)

*Brassica napus* BCH (NP_001302761.1)

100

100

80

99

99

62

50

76

0.05

**S9 Fig. Sequence analysis of CmBCH in 'CH' and 'FC'.**

Note: A: CmBCH was identical between 'CH' and 'FC'. B: Phylogenetic analysis of CmBCH.
