# Supplementary material for: Largely different carotenogenesis in two pummelo fruits with different flesh colors
Source: PLoS One. 2018 Jul 9;13(7):e0200320. doi: 10.1371/journal.pone.0200320 (PMC6037374; doi:10.1371/journal.pone.0200320)
Supplement: S10 Fig — A: CmZEPa and CmZEPb were detected in ‘CH’ and ‘FC’, respectively. A single amino acid difference in sequences was observed between CmZEPa and CmZEPb. B: Phylogenetic analysis of CmZEP. (DOC) [file pone.0200320.s010.doc]

A

B

*Prunus avium* ZEP (XP_021824846.1)

*Malus domestica* ZEP (AHA61555.1)

*Fragaria* x *ananassa* ZEP (AFR11774.2)

*Betula platyphylla* ZEP (ATE80670.1)

*Cucumis sativus* ZEP (NP_001292713.1)

*Populus trichocarpa* ZEP (XP_002307265.1)

*Hevea brasiliensis* ZEP (XP_021677792.1)

*Jatropha curcas* ZEP (XP_012079233.1)

*Citrus sinensis* ZEP (BAI79260.1)

*Citrus unshiu* ZEP (BAI79257.1)

**CmZEPb**

**CmZEPa**

*Citrus maxima* ZEP (ACE79170.1)

*Theobroma cacao* ZEP (EOX91418.1)

*Herrania umbratica* ZEP (XP_021274354.1)

*Bixa orellana* ZEP (AMJ39488.1)

*Carica papaya* ZEP (XP_021897642.1)

*Eutrema halophilum* ZEP (AAV85824.1)

*Brassica rapa* ZEP (ACM68704.1)

*Arabidopsis lyrata* ZEP (XP_002865032.1)

100

100

81

62

51

100

81

100

59

98

58

58

68

53

0.05

**S10 Fig. Sequences analysis of CmZEP in 'CH' and 'FC'.**

Note: A: CmZEPa and CmZEPb were detected in 'CH' and 'FC', respectively. A single amino acid difference in sequences was observed between CmZEPa and CmZEPb. B: Phylogenetic analysis of CmZEP.
