# Supplementary material for: Largely different carotenogenesis in two pummelo fruits with different flesh colors
Source: PLoS One. 2018 Jul 9;13(7):e0200320. doi: 10.1371/journal.pone.0200320 (PMC6037374; doi:10.1371/journal.pone.0200320)
Supplement: S11 Fig — A: CmNSY was identical between ‘CH’ and ‘FC’. B: Phylogenetic analysis of CmNSY. (DOC) [file pone.0200320.s011.doc]

A

>CmNSY

MVVAGIFIFAHSMAISSCLCHPLVLPLKDKPIGTWGNVGEDQRLTSAPRSMNAETFGRQITGVGTELRSEWSFLGGSRVIIRPQVTKFIRHQKGFHIHASWLSGPQLVSTVFTLGTAGVLPFYTLMVFAPKAELTKKSMESSIPYVVLGVLYAYLLYLSWTPDTLRLMFASKYWLPELPGIAKMFSSEITLASAWIHLLAVDLFAARHVFHDGLQNQIETRHSVSLCLLFCPIGILTHVITKALTKSS-

B

**CmNSY**

*Citrus sinensis* NSY (NP_001275861.1)

*Theobroma cacao* NSY (EOY04189.1)

*Bixa orellana* NSY (AMJ39493.1)

*Prunus persica* ABA DEFICIENT 4 (XP_007210532.1)

*Malus domestica* NSY (AEX97076.1)

*Prunus avium* ABA DEFICIENT 4 (XP_021827750.1)

*Prunus persica* ABA DEFICIENT 4 (XP_020415975.1)

*Cajanus cajan* ABA DEFICIENT 4 (XP_020222820.1)

*Vigna radiata* ABA DEFICIENT 4 (XP_014519230.1)

*Medicago truncatula* NSY (XP_013451304.1)

*Arachis ipaensis* ABA DEFICIENT 4 (XP_020977944.1)

*Medicago truncatula* NSY (XP_013447464.1)

*Vigna radiata* ABA DEFICIENT 4 (XP_014497321.1)

*Cajanus cajan* ABA DEFICIENT 4 (XP_020223996.1)

100

100

99

95

96

92

83

91

100

88

57

88

0.05

**S11 Fig. Sequence analysis of CmNSY in 'CH' and 'FC'.**

Note: A: CmNSY was identical between 'CH' and 'FC'. B: Phylogenetic analysis of CmNSY.
