# Supplementary material for: Largely different carotenogenesis in two pummelo fruits with different flesh colors
Source: PLoS One. 2018 Jul 9;13(7):e0200320. doi: 10.1371/journal.pone.0200320 (PMC6037374; doi:10.1371/journal.pone.0200320)
Supplement: S1 Table — The accession number was obtained from the Citrus sinensis (Valencia orange) annotation project (http://citrus.hzau.edu.cn/orange/). (DOC) [file pone.0200320.s012.doc]

**S1 Table. Primers used for quantitative real-time PCR (qRT-PCR) analysis.**

| **Gene** | **Forward (5’-3’)** | **Reverse (5’-3’)** | **References/ Accession number** |
| --- | --- | --- | --- |
| *Actin* | ATCTGCTGGAAGGTGCTGAG | CCAAGCAGCATGAAGATCAA | Liu *et al*., 2007 |
| *DXS* | GAAGCCGCAAAAAGCATCAC | CATCGACCTTTGCAGCAACTT | *Cs1g20530* |
| *DXR* | TGGTGGTCCTTTTGTCCTTC | GGCAAGCCTTGAATACACTG | *Cs5g05440* |
| *PSY* | GAGCAAGGATGCCTCAAATC | CCCGGACTGCTGTGTTTAAT | Liu *et al*., 2007 |
| *PDS* | CCTCTGTCGTCACTCGATCA | ATAATTGGCGGACAGGCATA | Liu *et al*, 2007 |
| *ZISO* | CATGGGTGTATTTCAGCGTGAT | TCCAAAGCAGCTGAAGCAGAA | *Cs5g24730.1* |
| *ZDS* | CCCTTGAGCATCCGCAAT | ATCAGTGCTCGTTGTATGCTTACTATATT | Liu *et al*, 2007 |
| *CRTISO* | TCATCCTCAAGCACAAAATGGT | TTCTTTCCATTCACATGGGTGTT | Liu *et al*., 2007 |
| *LCYb1* | CAGAATTGAGGCTTCGAACGA | GGCTATATGGTGGCAAGGACTT | Liu *et al*., 2007 |
| *LCYb2* | TGGCTCAACCAGGATGATCA | TTGGCCACAACCCATTCC | Guo *et al*., 2015 |
| *LCYe* | CAAGGAAACCGTGCCACATC | CAACTGGATATTGAGGGCATCA | Liu *et al*., 2007 |
| *BCH* | GGCACGTCGGCAATGG | TTTGGGATGGCCTACATGTTC | Liu *et al*., 2007 |
| *ZEP* | ACCGAGTCCCCAAGCAAAGT | GAAGCAATTCTTCGACGTGACA | Liu *et al*., 2007 |
| *NSY* | CTTCTTGCTTATGCCACCCTCTA | TGATCCACGCCAACATTACCC | *HM036683* |

Note: the accession number was obtained from the *Citrus sinensis* (Valencia orange) annotation project (http://citrus.hzau.edu.cn/orange/).
