# Supplementary material for: Largely different carotenogenesis in two pummelo fruits with different flesh colors
Source: PLoS One. 2018 Jul 9;13(7):e0200320. doi: 10.1371/journal.pone.0200320 (PMC6037374; doi:10.1371/journal.pone.0200320)
Supplement: S2 Table — The superscript ‘a’ gene accession means that the accession number was obtained from the NCBI database (http://www.ncbi.nlm.nih.gov/). The superscript ‘b’ genome accession means that the accession number was obtained from the Citrus sinensis (Valencia orange) annotation project (http://citrus.hzau.edu.cn/orange/). (DOC) [file pone.0200320.s013.doc]

**S2 Table. Primers used for gene cloning.**

| **Gene** | **Forward primer (5’-3’)** | **Reverse primers (5’-3’)** | **Accession number** |
| --- | --- | --- | --- |
| *DXR* | ATGGCATTGAATTTGCAGTC | TCATGCTGGAACAGGGCTT | *Cs5g05440^a^* |
| *PSY* | ATGTCTGTTGCATTGCTATGG | TTAAGCCTTACTGGTATATATTCTTG | *KP462726^b^* |
| *PDS* | ATGAGCCTTTGCTTCAGCG | TTAACACATGCTTGCCTCAGC | *Orange1.1t02361.1^a^* |
| *ZISO* | ATGAGCAGCAGTAGTTGTCTTCTT | CTACCAATGAAGCAGGAAACTG | *Cs5g24730.1^a^* |
| *CRTISO* | ATGTTCATCTCTTGTTGCTCTCT | TCATGCCAAACTCCTTAACC | *Cs6g13340.3^a^* |
| *LCYb1* | ATGGATACTTTACTCAAAACTCATAAC | TTAATCTGTATCTTGTACCAAGTTGT | *KP462729^b^* |
| *LCYb2* | ATGGCAACTCTTCTTAGCCC | TCAAATGGTTTCAAGGGCA | *AF169241^b^* |
| *LCYe* | ATGGAATACTACTGTCTTGGAGCTC | CTATAAAGTCAGGTATGTTCTTACC | *KP462730^b^* |
| *BCH* | ATGGCGGTCGGACTATTG | TTATTTTGGAACCCTGTTGTATG | *AF296158^b^* |
| *ZEP* | ATGGTTTCATCTATGTTCTACAATTC | TTACACTGCCTGAAGAATTTCAC | *Cs1g22620.1^a^* |
| *NSY* | ATGGTTGTTGCTGGGATTTTC | TCAAGAGCTTTTTGTCAGTGCTT | *HM036683^b^* |

Note: the superscript ‘a’ gene accession means that the accession number was obtained from the NCBI database (http://www.ncbi.nlm.nih.gov/). The superscript ‘b’ genome accession means that the accession number was obtained from the *Citrus sinensis* (Valencia orange) annotation project (http://citrus.hzau.edu.cn/orange/).
